# Bonding State and Thermal Expansion Coefficient of Mn-Doped Ba_0.5_Sr_0.5_FeO_3−δ_ Perovskite Oxides for IT-SOFCs

**DOI:** 10.3390/nano14010082

**Published:** 2023-12-27

**Authors:** Taeheun Lim, Sung-sin Yun, Kanghee Jo, Heesoo Lee

**Affiliations:** 1 School of Materials Science and Engineering, Pusan National University, Busan 46241, Republic of Korea; taeheunlim@pusan.ac.kr (T.L.); ssyun@dongjin.com (S.-s.Y.); jokanghee@pusan.ac.kr (K.J.); 2 Electronic Materials Business Unit II Manufacturing Technology Team, Dongjin Semichem Co., Ltd., Incheon 22824, Republic of Korea

**Keywords:** cobalt-free cathode, oxygen vacancy, oxygen reduction reaction, area-specific resistance, thermal expansion coefficient

## Abstract

The oxygen vacancy formation behavior and electrochemical and thermal properties of Ba_0.5_Sr_0.5_Fe_1−x_Mn_x_O_3−δ_ (BSFMnx, x = 0–0.15) cathode materials were investigated. For thermogravimetric analysis, the weight decreased from 1.98% (x = 0) to 1.81% (x = 0.15) in the 400–950 °C range, which was due to oxygen loss from the lattice. The average oxidation state of the B-site increased, the O_ads_/O_lat_ ratio decreased, and the binding energy of the O_lat_ peak increased with Mn doping. These results indicate that Mn doping increases the strength of the metal–oxygen bond and decreases the amount of oxygen vacancies in the lattice. The electrical conductivity of BSFMnx increased with the temperature due to the thermally activated small-polaron hopping mechanism showing a maximum value of 10.4 S cm^−1^ (x = 0.15) at 450 °C. The area-specific resistance of BSFMn0.15 was 0.14 Ω cm^2^ at 700 °C and the thermal expansion coefficient (TEC) gradually decreased to 12.7 × 10^−6^ K^−1^, which is similar to that of Ce_0.8_Sm_0.2_O_2_ (SDC) (12.2 × 10^−6^ K^−1^). Mn doping increased the metal–oxygen bonding energy, which reduced the oxygen reduction reaction activity but improved the electrical conductivity and thermal stability with SDC.

## 1. Introduction

Solid oxide fuel cells (SOFCs) have attracted considerable attention because they convert chemical energy directly into electrical energy in a clean and high-efficiency manner [1,2,3]. Nevertheless, the high operating temperature (800–1000 °C) of SOFCs results in many problems, such as challenges with material compatibility and short life spans [4,5]. Recently, substantial efforts have been devoted to the development of intermediate temperature SOFCs (IT-SOFCs), which operate at intermediate temperatures (600–800 °C), to overcome these challenges [6,7]. However, lowering the operating temperature increases the electrode polarization resistance and degrades the electrocatalytic activity of materials [8,9]. The main contribution to the polarization resistance comes from the cathode due to a sluggish oxygen reduction reaction (ORR) [10]. Therefore, the development of a cathode material with high ORR activity is required for the application of IT-SOFCs [11].

Mixed ionic and electronic conducting (MIEC) oxides have been considered as cathode materials because of their superior ORR activity at intermediate temperatures [12,13,14]. Many cobalt-containing MIEC oxides, such as La_0.6_Sr_0.4_CoO_3−δ_, La_0.6_Sr_0.4_Co_0.2_Fe_0.8_O_3−δ_, and Ba_0.5_Sr_0.5_Co_0.8_Fe_0.2_O_3−δ_, have been used as cathode materials in IT-SOFCs [15,16,17]. However, these cobalt-containing cathodes have durability and reliability issues due to the high redox activity of cobalt, their high thermal expansion coefficient (TEC), and their low chemical stability [18,19]. Therefore, it is desirable to develop cobalt-free cathodes with good electrocatalytic activity and thermal stability for IT-SOFCs.

Numerous efforts have been made to develop iron-based MIEC cathodes such as BaFeO_3−δ_ (BFO) [20]. Among the iron-based cathode materials, cubic BFO materials exhibit good mixed oxygen–electron conduction and excellent chemical and thermal stabilities compared with those of cobalt-based materials [21]. Cubic BFO exhibits high oxygen ion conduction owing to the presence of disordered oxygen vacancies and three-dimensional oxygen diffusion paths [22]. However, pristine BFO has several crystal structures, which depend on the temperature, atmosphere, and oxygen vacancies in the lattice [23,24]. To stabilize cubic-phase BFO, several studies have doped the A- and/or B-sites with cations, which improve the cathode’s properties [25]. Dong et al. stabilized the cubic phase by doping La into the A-site of BFO and obtained a low area-specific resistance (ASR) of 0.021 Ω cm^2^ at 700 °C [23]. Zhao et al. stabilized the cubic phase by doping Sr and Cu into the A- and B-sites of BFO, respectively, and obtained a low ASR of 0.137 Ω cm^2^ at 700 °C [26]. 

Many studies have also been published on the effect of Mn doping into the B-site of perovskite oxides and the properties of Mn-based perovskite oxides. Olsson et al. lowered the thermal expansion coefficient by Mn doping in Sm_0.75_A_0.25_Co_1−x_Mn_x_O_2.88_ (A = Ca, Sr; x = 0.125, 0.25) [27]. Świerczek et al. [28] and Klimkowicz et al. [29] discussed the oxygen storage capacity along with conducting a crystal structure analysis in the BaErMn_2_O_5_–BaErMn_2_O_6_ system and BaY_1−x_Pr_x_Mn_2_O_5+δ_, respectively.

In this study, the oxygen vacancy formation behavior of Ba_0.5_Sr_0.5_Fe_1−x_Mn_x_O_3−δ_ (BSFMnx, x = 0, 0.05, 0.1, 0.15) and its electrical conductivity, polarization resistance, and thermal expansion changes were investigated. Crystal structure analysis and thermogravimetric analysis (TGA) of BSFMnx were performed, and the electronic structure was analyzed using X-ray photoelectron spectroscopy (XPS). The electrical conductivity of BSFMnx was measured and its TEC was calculated to confirm its thermal stability with the electrolyte. The changes in the electrochemical properties of the cathode were observed by measuring the ASR using impedance spectra. 

## 2. Experimental Procedure

Ba_0.5_Sr_0.5_Fe_1−x_Mn_x_O_3−δ_ (x = 0, 0.05, 0.10, 0.15) powders were synthesized via a solid-state reaction. Stoichiometric amounts of BaCO_3_ (99.9% purity; Sigma-Aldrich, St. Louis, MO, USA), SrCO_3_ (99.9% purity; Sigma-Aldrich), Fe_2_O_3_ (99.9% purity; Sigma-Aldrich), and MnO_2_ (99.9% purity; Alfa Aesar, Haverhill, MA, USA) powders were weighed and mixed. The mixture was ball-milled in a polyethylene container with ethanol and zirconia balls for 24 h, dried at 100 °C for 12 h, and calcined at 1300 °C for 10 h. The calcined powders were ground and sieved using a 250 μm mesh. Table 1 shows the abbreviations of the Ba_0.5_Sr_0.5_Fe_1−x_Mn_x_O_3−δ_ materials according to their Mn content.

Symmetric cells (BSFMnx|SDC|BSFMnx) were prepared to investigate the electrochemical properties of the BSF, BSFMn0.05, BSFMn0.10, and BSFMn0.15 cathodes supported on Samarium-doped Ceria (Ce_0.8_Sm_0.2_O_2_, SDC; Fuel Cell Materials, Lewis Center, OH, USA) electrolyte pellets. The pellets were sintered at 1500 °C for 10 h to obtain a diameter of 17 mm and then polished to a thickness of 600 μm. BSFMnx powder was mixed with a vehicle (Fuel Cell Materials) to prepare BSFMnx pastes using a three-roll mill, and these pastes were screen-printed on both sides of the SDC pellets with an area of 0.2826 cm^2^. After drying, the symmetric cells were sintered at 1100 °C for 2 h in air.

The X-ray diffraction (XRD) patterns of the synthesized powders were recorded at room temperature using a step scan procedure (0.02°/2θ step, 1° min^−1^) in the 2θ range 20°–80° (X’pert PRO-MPD, λ = 1.54 Å). The structural parameters were obtained by Rietveld refinement of the XRD patterns using PANalytical X’Pert HighScore Plus software ’version 3.0c(3.0.3). The lattice spacing of BSFMnx was calculated using Digital Micrograph software version 3.9.1 (Gatan, Pleasanton, CA, USA) from high-resolution transmission electron microscopy (HR-TEM, JEOL JEM-2100F, JEOL, Tokyo, Japan) images. Thermogravimetric analysis (TGA) was performed using a thermal analyzer (NETZSCH STA 409 PC/PG, NETZSCH-Gerätebau GmbH, Selb, Germany) to confirm the oxygen vacancy formation temperature and weight reduction in BSFMnx. An approximately 100 mg BSFMnx sample was heated from room temperature to 950 °C in a nitrogen atmosphere at a heating rate of 5 °C min^−1^. XPS (Thermo Fisher Scientific, Waltham, MA, USA) was performed to measure the oxidation state of BSFMnx. The XPS spectra were calibrated using a C 1s signal at 284.6 eV. 

The electrical conductivity of BSFMnx was measured using a 4-probe DC technique (ISO 23331:2021) in the range of 300–900 °C, and Pt wires were wrapped around the sintered bars with dimensions of 5 × 3 × 30 mm^3^. A direct current of 50 mA was supplied from a current source (Keithley 2400, Solon, OH, USA), and the corresponding voltage drop was collected using a multimeter (Agilent, 34401A, Santa Clara, CA, USA). The electrical conductivity was calculated using the following equation:

(1) $$\sigma =\frac{L}{R\times A}=\frac{I\times L}{V\times A}$$

where *σ* is the conductivity, *R* is the resistance, *V* is the measured voltage, *I* is the current, *L* is the distance between the voltage sensing electrodes, and *A* is the measured cross-sectional area of the sample.

The TEC of BSFMnx was measured using a dilatometer (Netzsch DIL 402C, Netzsch, Selb, Germany) in the temperature range 25–940 °C in air at a heating rate of 5 °C min^−1^. Electrochemical impedance spectroscopy (EIS) was performed using an Iviumstat (Ivium, The Netherlands) instrument over the frequency range of 10^6^ to 10^−2^ Hz at 700 °C, with an excitation voltage of 10 mV.

## 3. Results and Discussion

**Figure 1:**
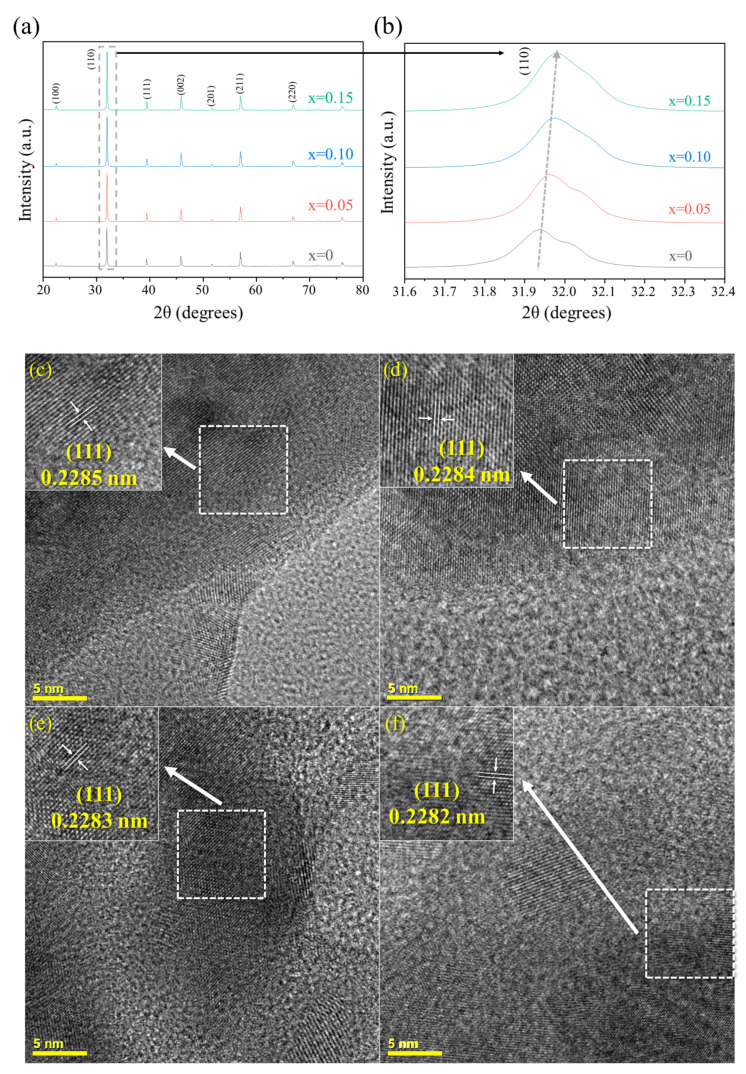
(**a**) XRD patterns of Ba_0.5_Sr_0.5_Fe_1−x_Mn_x_O_3−δ_ (x = 0, 0.05, 0.10, 0.15) and (**b**) enlarged (110) peaks. HR-TEM images of (**c**) BSF, (**d**) BSFMn0.05, (**e**) BSFMn0.10, and (**f**) BSFMn0.15.

**Figure 2:**
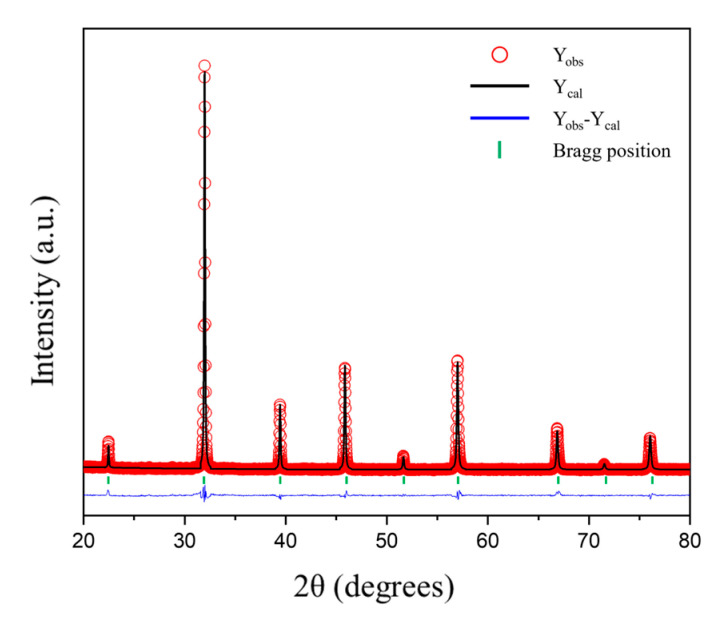
Rietveld refinement result of Ba_0.5_Sr_0.5_Fe_1−x_Mn_x_O_3−δ_ after calcination at 1300 °C for 10 h in air. The open symbol represents the observed intensities, the black line is the calculated intensities, the blue line is the difference between the observed and calculated intensities, and the green vertical bars are the Bragg positions.

Figure 1a shows the XRD patterns of the BSFMnx (x = 0, 0.05, 0.10, 0.15) powder sintered at 1300 °C for 10 h. No secondary phases appeared in the patterns. The results of Rietveld refinement (Table 2 and Figure 2) indicate that all the BSFMnx compositions crystallized into a cubic perovskite structure with space group $Pm\bar{3}m$. As shown in Figure 1b, the 2θ value of the (110) peak shifted to higher angles, and the lattice constant decreased as Mn doping increased. Both Fe and Mn can take multivalent states, and the ionic radius of Mn^4+^ (0.53 Å) is smaller than that of Fe^4+^ (0.585 Å), whereas Mn^3+^ and Fe^3+^ possess the same ionic radius (0.645 Å) with sixfold coordination [30,31]. Therefore, the lattice constant decreases as Mn doping increases. Figure 1c–f display HR-TEM images of the BSFMnx samples, and the lattice fringe spacing is approximately 0.228 nm, which corresponds to the (111) plane of BSFMnx [32]. The lattice spacing of the (111) plane decreased from 0.2285 to 0.2282 nm as the Mn doping increased, which corresponds to that calculated using the Bragg equation given in Equation (2) (d111 = 0.2285 nm (x = 0), 0.2284 nm (x = 0.05), 0.2283 nm (x = 0.10), and 0.2282 nm (x = 0.15)).

(2) $$a\;cubic\;structure,\;d_{hkl}=\frac{a}{\sqrt{h^{2}+k^{2}+l^{2}}}$$

**Figure 3:**
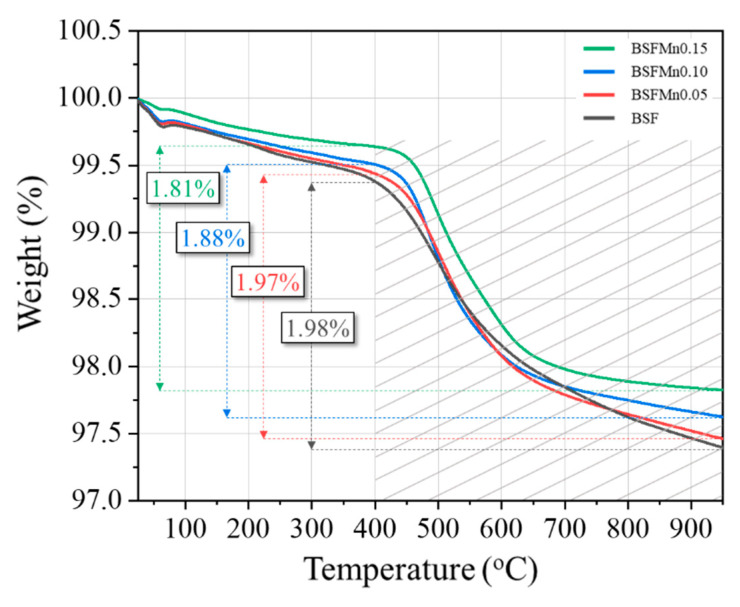
Thermogravimetric (TG) curves of Ba_0.5_Sr_0.5_Fe_1−x_Mn_x_O_3−δ_ (x = 0, 0.05, 0.10, 0.15).

Thermogravimetric analysis was used to confirm the effect of Mn doping on oxygen vacancy formation, and Figure 3 shows the TG curve of the BSFMnx samples. In order to more clearly observe the formation of oxygen vacancies according to the Mn content, this was measured in a reducing atmosphere using nitrogen. The rate of weight loss of the BSFMnx samples decreased with increasing Mn content. This weight loss was due to oxygen loss from the crystal lattice and is consequently related to the formation of oxygen vacancies corresponding to the reduction in B-site transition metal ions [33].

There was a weight loss of less than 1% during heating from room temperature to 400 °C, which is thought to be due to the desorption of physically adsorbed moisture and carbon dioxide [34]. In the 400–995 °C temperature range, the slopes of the TG curves changed and the weight decreased by 1.98% (x = 0), 1.97% (x = 0.05), 1.88% (x = 0.10), and 1.81% (x = 0.15), respectively. Weight loss in this temperature range is due to the loss of lattice oxygen. The degree of oxygen loss decreases as the Mn content increases, which indicates that oxygen vacancies are more difficult to form with Mn doping. 

**Figure 4:**
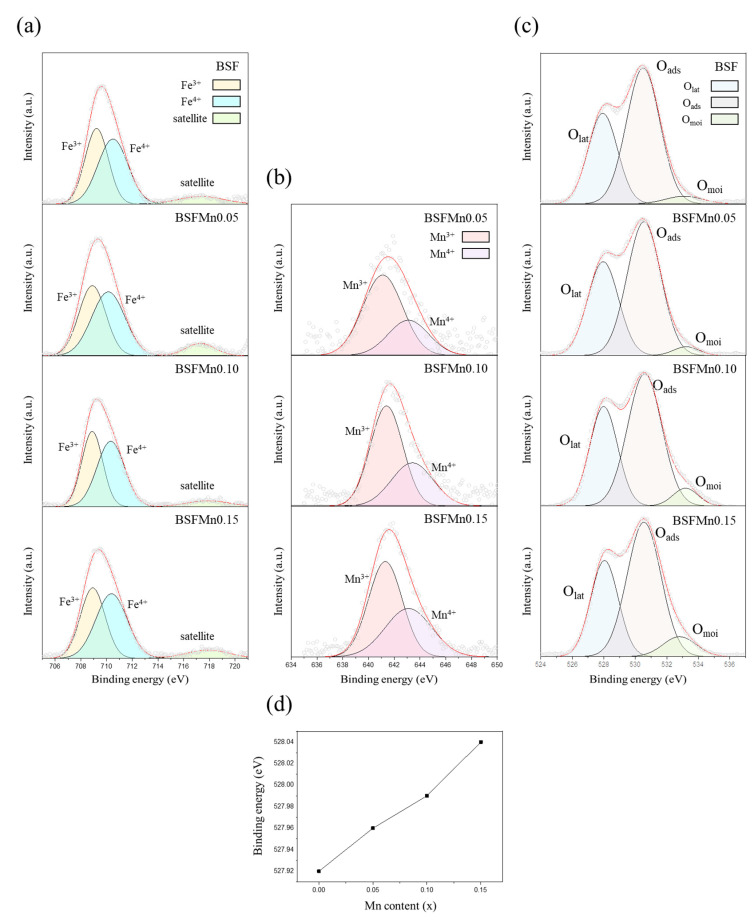
(**a**) Fe 2p_3/2_, (**b**) Mn 2p_3/2_, and (**c**) O 1s XPS spectra of Ba_0.5_Sr_0.5_Fe_1−x_Mn_x_O_3−δ_ (x = 0, 0.05, 0.10, 0.15). (**d**) Binding energy of the lattice oxygen (O_lat_) peak as a function of Mn content.

The XPS spectra of Fe 2p_3/2_ were divided into two parts at approximately 708.9 eV (Fe^3+^) and 710.3 eV (Fe^4+^), with weak satellite shake-up peaks at 717.1–717.9 eV (Figure 4a) [35,36,37]. The Mn 2p_3/2_ spectra exhibited two distinct peaks at approximately 641.3 eV (Mn^3+^) and 643.3 eV (Mn^4+^), as shown in Figure 4b [38,39]. With increasing Mn doping, the Fe^4+^/Fe^3+^ and Mn^4+^/Mn^3+^ ratios gradually increased. Hence, Mn doping increased the average oxidation state of the B-site from +3.5170 (x = 0) to 3.5208 (x = 0.15), as listed in Table 3, which indicates that the M–O (M = Fe, Mn) bond strength increased [40,41]. 

As shown in Figure 4c, the O 1s spectra were fitted with three different components at approximately 527.9, 530.5, and 533 eV, corresponding to lattice oxygen (O_lat_), adsorbed oxygen (O_ads_), and moisture on the surface (O_moi_), respectively [36,42]. The adsorbed oxygen can easily be released from the surface of the crystal lattice with the increase in temperature and can lead to the formation of oxygen vacancies. The ratio of O_ads_/O_lat_ calculated from the area under the corresponding XPS peaks can be regarded as a criterion for comparing the oxygen vacancy concentration [43,44]. The O_ads_/O_lat_ ratio decreased from 1.71 (x = 0) to 1.62 (x = 0.15), indicating that the amount of oxygen vacancies decreased with increasing Mn doping. It can be expected that the O_lat_ ratio will gradually increase. However, as explained earlier, the decrease in oxygen vacancy content can be discussed in terms of the O_ads_/O_lat_ ratio. Therefore, we believe that the O_lat_ value alone, which does not clearly depend on the Mn content, does not have much significance regarding the possibility of oxygen vacancy formation.

Figure 4d shows that the binding energy of the O_lat_ peak increased from 527.92 eV (x = 0) to 528.04 (x = 0.15) with increasing Mn doping. The shift of the O_lat_ peak to a higher binding energy is due to increased M-O (M = Fe, Mn) bond strength, which means that the formation of oxygen vacancies becomes more difficult [45].

**Figure 5:**
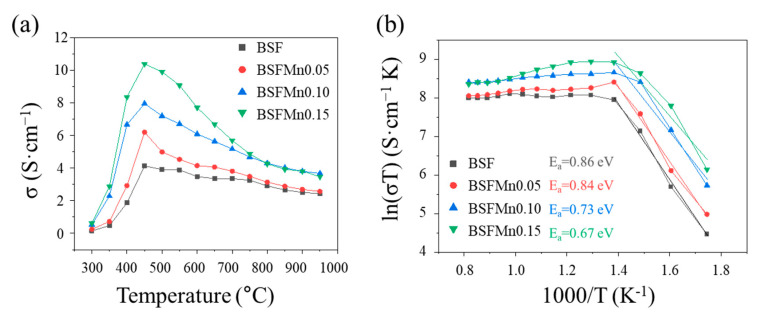
(**a**) The temperature dependence of the electrical conductivity of Ba_0.5_Sr_0.5_Fe_1−x_Mn_x_O_3−δ_ (x = 0, 0.05, 0.10, 0.15), measured from 300 °C to 950 °C in air; (**b**) ln(σT) vs. 1000/T plot.

Fe-based perovskite oxides have mixed ionic–electronic conductivity because of the simultaneous presence of oxygen vacancies and electron holes as charge carriers. However, Figure 5 mainly shows the electrical conductivity because this is about two orders of magnitude higher than the ionic conductivity [46,47]. As the amount of Mn doping increased, the overall electrical conductivity also increased. Among the cathodes, the BSFMn0.15 cathode showed the highest electrical conductivity at 450 °C in air at 10.4 S cm^−1^. The maximum conductivity was obtained at 450 °C for all compositions; after that, their conductivity decreased as the temperature increased. 

Clearly, BSFMnx confirmed that the conduction mechanism changes from semiconductor-like behavior to metal-like behavior over 450 °C regardless of the Mn doping content. At the same temperature, the higher the Mn doping amount, the higher the electrical conductivity. This improvement is due to the number of Me^4+^ and Me^3+^ pairs (Me^4+^–O–Me^3+^ bonds, Me = Fe and Mn) contributing to the increase in small-polaron hopping with increasing Mn content.

The decrease in electrical conductivity is related to the breakdown of (Fe, Mn)–O–(Fe, Mn) bonds above 450 °C because the oxygen vacancies are formed and the charge carrier concentration is reduced due to the reduction of Fe^4+^ and Mn^4+^ (Equation (3)), which is in good agreement with the thermogravimetric results.

(3) $$2M_{M}^{\times }\left(Fe^{4+},\;Mn^{4+}\right)+O_{O}^{\times }\;↔\;2M_{M}^{\prime }\left(Fe^{3+},\;Mn^{3+}\right)+V_{O}^{{}^{..}}+\frac{1}{2}O_{2}\;\left(M=Fe,\;Mn\right)$$

Figure 5b shows an Arrhenius plot of the electrical conductivity of BSFMnx, and the relationship between electrical conductivity (*σ*) and temperature follows the Arrhenius equation:

(4) $$\sigma =Aexp\left(-\frac{E_{a}}{kT}\right)$$

where *A*, *E_a_*, *k*, and *T* are the pre-exponential constant, activation energy, Boltzman constant, and temperature, respectively. According to the slope of the linear fit over temperatures of 300–450 °C, the activation energy (*E_a_*) was 0.86 eV ± 0.004 eV (x = 0), 0.84 eV ± 0.005 eV (x = 0.05), 0.73 eV ± 0.008 eV (x = 0.10), and 0.67 eV ± 0.014 eV (x = 0.15). A low activation energy value helps to enhance the hopping of charge carriers, thereby increasing the electrical conductivity.

**Figure 6:**
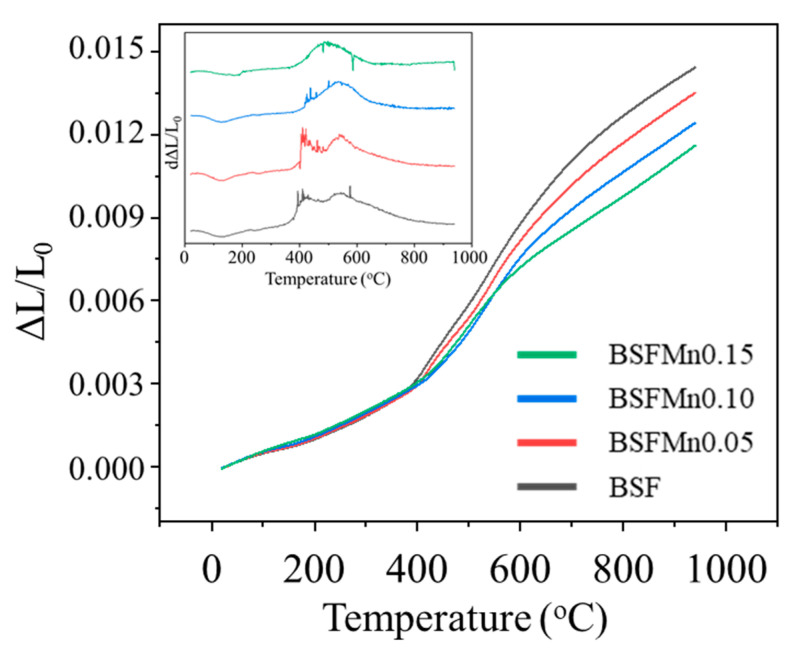
Thermal expansion (ΔL/L_0_) curves of Ba_0.5_Sr_0.5_Fe_1−x_Mn_x_O_3−δ_ (x = 0, 0.05, 0.10, 0.15). The inset shows the first derivative of ΔL/L_0_.

The TEC of the cathode and electrolyte should match to ensure the long-term stability of the SOFC system under thermal cycling [48]. Figure 6 depicts the thermal expansion curves of the BSFMnx samples in the temperature range 25–940 °C, and the TECs for different sections of that temperature range are listed in Table 4. In the 25–940 °C range, the TEC gradually decreased from 15.7 × 10^−6^ K^−1^ (x = 0) to 12.7 × 10^−6^ K^−1^ (x = 0.15), which is similar to that of SDC (12.2 × 10^−6^ K^−1^) [49]. The TEC of BSFMn0.15 is lower than that of cobalt-containing cathodes, such as La_0.6_Sr_0.4_CoO_3−δ_ (20.5 × 10^−6^ K^−1^, 30–1000 °C), La_0.6_Sr_0.4_Co_0.2_Fe_0.8_O_3−δ_ (17.5 × 10^−6^ K^−1^, 30–1000 °C), and Ba_0.5_Sr_0.5_Co_0.8_Fe_0.2_O_3−δ_ (19.7 × 10^−6^ K^−1^, 50–900 °C) [50,51,52].

**Figure 7:**
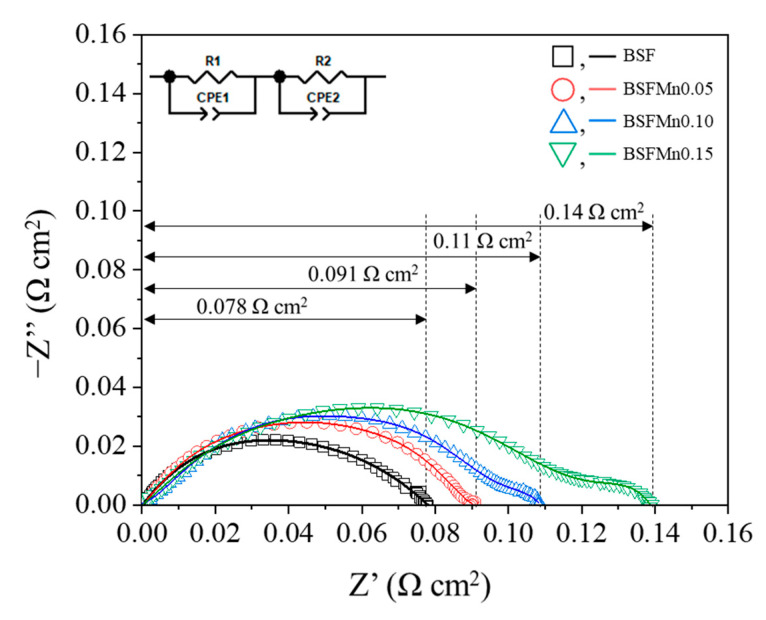
Nyquist plots of the impedance spectra obtained from Ba_0.5_Sr_0.5_Fe_1−x_Mn_x_O_3−δ_ (x = 0, 0.05, 0.10, 0.15) symmetric cells at 700 °C. The observed data and fitted data are shown as dotted and solid lines.

Inflections were observed in the BSFMnx curves at 400 °C because of the formation of oxygen vacancies, as shown in Equation (2), and lattice expansion occurred along with the reduction of Fe^4+^ and Mn^4+^ [53,54]. The inset in Figure 7 shows the first derivative of the thermal expansion curve, where the inflections are observed above 400 °C due to the formation of oxygen vacancies. As Mn doping increased, the temperature of the inflection point—that is, the oxygen vacancy formation temperature—increased. It can be determined that the average oxidation state of B-site ions and the binding energy of O_lat_ increased with increasing Mn doping, which increased both M–O (M = Fe, Mn) bond strength and the difficulty of oxygen vacancy formation in the lattice. Therefore, BSFM0.15 cathodes can represent potential cathodes for IT-SOFCs due to their electrical conductivity, ASR value, and thermal stability with SDC.

The ORR catalytic activity of BSFMnx cathodes was assessed via electrochemical impedance spectroscopy (EIS) of a symmetric cell configuration (BSFMnx|SDC|BSFMnx), and the EIS plots measured at 700 °C in air are shown in Figure 6. The intercepts on the real axis of the EIS curves represent the ASR, which is the non-ohmic resistance of the electrodes [11]. The overall ASR divided by two is the polarization resistance of one cathode with the electrolyte. The ASR values of the BSFMnx cathodes increased with increasing Mn doping: 0.078 Ω cm^2^ (x = 0), 0.091 Ω cm^2^ (x = 0.05), 0.11 Ω cm^2^ (x = 0.10), and 0.14 Ω cm^2^ (x = 0.15). The reduction in the oxygen ion conductivity of BSFMnx is attributed to the higher M–O (M = Fe, Mn) bond strength, resulting in decreased oxygen vacancies. However, the ASR values of the BSFMnx cathode are lower than those of other cobalt-free cathode materials: Ba_0.5_Sr_0.5_Zn_0.2_Fe_0.8_O_3–δ_ (0.23 Ω cm^2^ at 700 °C), SrFe_0.9_Nb_0.1_O_3−δ_ (0.29 Ω cm^2^ at 700 °C), and La_0.5_Sr_0.5_FeO_3−δ_ (0.79 Ω cm^2^ at 700 °C) [54,55,56]. The ASR value of BSFMnx was relatively lower than that of cobalt-free cathodes, because as electrical conductivity increases, the ORR activity of the cathode also improves [57].

## 4. Conclusions

We investigated the relationship between oxygen vacancy formation in BSFMnx by Mn doping and its electrical conductivity, area-specific resistance, and thermal expansion coefficient. BSFMnx crystallized into a cubic perovskite structure with the space group $Pm\bar{3}m$ and Rietveld refinement confirmed that the lattice constant decreased with increasing Mn doping. The weight loss was 1.81% (x = 0.15) in the temperature range of 400–950 °C, a decrease of about 10% compared to 1.98% (x = 0). This weight loss was due to oxygen loss from the lattice, and it was confirmed that oxygen vacancy formation decreased as Mn doping increased. The average oxidation state of the B-site increased from +3.5170 (x = 0) to +3.5208 (x = 0.15), and the O_ads_/O_lat_ ratio decreased from 1.71 (x = 0) to 1.62 (x = 0.15). The binding energy of the O_lat_ peak increased from 527.92 eV (x = 0) to 528.04 (x = 0.15) with increasing Mn doping. These results indicate that Mn doping increases the strength of the metal–oxygen bonds and decreases the amount of oxygen vacancies in the lattice. BSFMn0.15 showed the highest electrical conductivity of 10.4 S cm^−1^ at 450 °C. The ASRs of symmetric BSFMnx cells were 0.078 Ω cm^2^ (x = 0) and 0.14 Ω cm^2^ (x = 0.15) at 700 °C, which are lower than those of other cobalt-free materials (0.23–0.79 Ω cm^2^). The TEC of BSFMnx gradually decreased from 15.8 × 10^−6^ K^−1^ (x = 0) to 12.7 × 10^−6^ K^−1^ (x = 0.15), which is similar to that of SDC (12.2 × 10^−6^ K^−1^), the electrolyte. It can be determined that Mn doping increases the metal–oxygen bond strength and the difficulty of oxygen vacancy formation in the lattice but improves the electrical conductivity and thermal stability with SDC.

## Figures and Tables

**Table 1:** Compositions and abbreviations of the Ba_0.5_Sr_0.5_Fe_1−x_Mn_x_O_3−δ_ (x = 0, 0.05, 0.10, 0.15) samples.

Mn Content	Composition	Abbreviation
x = 0	Ba_0.5_Sr_0.5_FeO_3−δ_	BSF
x = 0.05	Ba_0.5_Sr_0.5_Fe_0.95_Mn_0.05_O_3−δ_	BSFMn0.05
x = 0.10	Ba_0.5_Sr_0.5_Fe_0.9_Mn_0.1_O_3−δ_	BSFMn0.10
x = 0.15	Ba_0.5_Sr_0.5_Fe_0.85_Mn_0.15_O_3−δ_	BSFMn0.15

**Table 2:** Structural parameters of Ba_0.5_Sr_0.5_Fe_1−x_Mn_x_O_3−δ_ (x = 0, 0.05, 0.10, 0.15) sintered at 1300 °C, calculated by Rietveld refinement of the room-temperature XRD data.

Parameters	Composition			
	BSF	BSFMn0.05	BSFMn0.10	BSFMn0.15
a = b = c [Å]	3.95714 (3)	3.95527 (4)	3.95428 (7)	3.95321 (5)
Volume [Å^3^]	61.96469 (5)	61.87688 (3)	61.83043 (5)	61.78025 (5)
Structure	$Pm\bar{3}m$	$Pm\bar{3}m$	$Pm\bar{3}m$	$Pm\bar{3}m$
R_wp_ [%]	3.97668	4.77116	3.94348	3.45366
R_exp_ [%]	1.9525	1.83542	1.82135	1.78808
χ^2^	4.14819	6.75734	4.68784	3.73067

**Table 3:** Fitting results of the Fe 2p_3/2_, Mn 2p_3/2_, and O 1s XPS spectra of Ba_0.5_Sr_0.5_Fe_1−x_Mn_x_O_3−δ_ (x = 0, 0.05, 0.10, 0.15).

	Fe^3+^	Fe^4+^	Mn^3+^	Mn^4+^	Average Oxidation State	δ_0_	O_lat_	O_ads_	O_moi_	O_ads_/O_lat_
BSF	48.3%	51.7%	-	-	+3.5170	0.2415	35.51%	60.76%	3.73%	1.71
BSFMn0.05	47.1%	52.9%	70.0%	30.0%	+3.5176	0.2412	35.10%	59.60%	4.60%	1.66
BSFMn0.10	46.4%	53.6%	64.0%	36.0%	+3.5184	0.2408	35.81%	58.42%	5.77%	1.63

**Table 4:** TECs (10^−6^ K^−1^) of Ba_0.5_Sr_0.5_Fe_1−x_Mn_x_O_3−δ_ (x = 0, 0.05, 0.10, 0.15) at various temperature ranges.

	700 °C	400–600 °C	600–800 °C	20–940 °C
BSF	19.0	27.9	19.5	15.7
BSFMn0.05	17.8	26.2	17.7	14.8
BSFMn0.10	15.0	23.1	15.5	13.6
BSFMn0.15	12.1	20.8	12.8	12.7

## Data Availability

Data available upon reasonable request.
